# Lean project planning – Bridging last planner system and earned value management

**DOI:** 10.1016/j.heliyon.2024.e37810

**Published:** 2024-09-11

**Authors:** Jan Emblemsvåg

**Affiliations:** Norwegian University of Science and Technology (NTNU), Ålesund, Norway

**Keywords:** Fabrication, Lean, Project scheduling, Portfolio management, Pressure vessel

## Abstract

Earned Value Management (EVM) has become a *de facto* standard for project planning after decades of applications worldwide, and most industry contracts that require some of the reporting features of EVM. However, EVM has shortcomings such as too early release of work and no process perspective to facilitate improvements. The Last Planner System (LPS) was developed to overcome the shortcomings of EVM utilizing insights from lean manufacturing. Unfortunately, LPS has not reached the same level of acceptance as EVM. A new approach, Lean Project Planning (LPP), is developed to take advantage of the strengths of both approaches. In this paper, LPS and EVM are compared to each other, and the resulting LPP is presented. The paper discusses a LPP implementation across the project portfolio at a Norwegian pressure vessel fabrication company. The results show that LPP provided accurate Estimate At Completion (EAC) for both costs and delivery times despite taking on many major Variation Orders. The approach is arguably a step forward for turning plans into planning in project-based industries such as shipbuilding and fabrication of pressure vessels. The complexity of pressure vessel fabrication stress-tested the LPP, and some improvements were identified for future work.

## 1 Introduction

The only source of knowledge is experience.

Albert Einstein

In 2017, the Project Management Institute (PMI) estimated that the value of project-oriented economic activity worldwide would grow from $12 trillion in 2017 to $20 trillion in 2027, in the process putting some 88 million people to work in project management–oriented roles and in the process displacing Operations as the main economic engine [1]. While only 35 % of projects globally can be considered ‘successful,’ statistics vary by industry. For example, the United States Government Accountability Office performed a study of 778 major Information Technology (IT) projects performed in fiscal year 2008 by the 24 major agencies of the US Government. The findings were quite typical of many IT projects. Powner [2] reported that 53 % of these projects (413) totaling 25.2 billion USD either were poorly planned (79 %), performed poorly (15 %) or both (6 %).

These outcomes mean that improving project planning and control is important for many companies. One of the most recognized tools is the Earned Value Management (EVM) method [3]. EVM includes valuable metrics such as the Cost Performance Index (CPI) and the Schedule Performance Index (SPI), which enable relatively accurate prediction of Estimate At Completion (EAC) after just 15 %–20 % project completion [4]. However, Yong-Woo and Ballard [5] have shown that EVM suffers from the assumptions that activities and cost accounts are independent and that “… making BCWP (earned-value) a priority in releasing assignments to the field which prevents quality assignments, which in turn results in unreliability of work flow”. Fleming and Koppelman [4] acknowledge that EVM can be too complicated for many companies. Yet, government contracts require the usage of EVM in larger projects, see Ref. [6]. Emblemsvag [7] reports a number of dysfunctional behaviors in other industries. In addition, de Souza and de Souza [8] report negative influence on project quality.

In parallel, the successful lean manufacturing principles have been refined to the realities of project-based industries resulting in Lean Construction (LC). LC came from the realization that “… current project management attempts to manage by scheduling, cost and output measures, …are often not effective. By contrast, lean construction attempts to manage the value created by all the work processes used between project conception and delivery” [9]. A central element in LC is the Last Planner System (LPS), which significantly improves productivity in projects [10]. In one case “project productivity improved by 86 % in consequence of this improvement in work flow reliability” [11]. Unfortunately, LPS also has issues related to contract compliance and reporting [12]. LPS simply is not recognized as a contractually agreeable approach in project-based industry. For example, EVM, or EVM-similar, approaches were required in all of the roughly 100 contracts this author has been involved in. Thus, research is required to improve the situation.

### 1.1 The research questions

Both approaches have useful attributes and shortcomings. The question is whether or not the two approaches can be combined to reap the best of both, and also avoid the shortcomings of both. This has been attempted before in shipbuilding, but only to a limited extent as a crucial element of EVM was left out – the usage of dependencies between activities to enable accurate forecasting, (see Ref. [12]). Dependencies are difficult to operationalize realistically leading to extra costs and longer durations, which is unacceptable in highly competitive bidding situations [12].

This paper therefore proposes two research questions. The first is: can EVM and LPS be combined to overcome the shortcomings of both approaches while reaping positive synergies? The second research question is: can such a combination of EVM and LPS be successfully applied in an industry with more stringent requirements than shipbuilding such as the design and fabrication of advanced pressure vessels?” The combined approach was labeled Lean Project Planning (LPP).

The stringent requirements of pressure vessel fabrication represent a challenge, and is therefore good for stress-testing LPP. First, this type of fabrication has a key challenge, similar to manufacturing, in that pressure vessels of various projects move through the fabrication at the same time and frequently share resources. Hence, the entire project portfolio must be simultaneously planned on an operational level, and not just capacity planning which is common in shipbuilding. Second, unlike a ship where many people can work at the same time, on pressure vessels only a handful of people can work at the same time. Thus, adding extra capacity to recuperate losses in progress, which is common in shipbuilding and civil construction, is possible only to a minor degree. Third, the products are genuinely unique products delivered through a project-based execution model, which effectively inhibits the traditional manufacturing production planning approach found in Enterprise Resource Planning (ERP) systems using status codes as execution progresses.

Thus, the novelty in this research lies in combining the best of two approaches (EVM and LPS) and testing the new approach (LPP) in a very demanding setting where the entire project portfolio must be planned operationally on a detailed level, like in manufacturing, but all the products are unique and are fabrication in a project-based business model mode with very limited possibility of recuperating progress losses by adding more capacity.

The answer to both of these research questions provides practical insights and scientific knowledge on two levels. The first level will resolve the aforementioned weaknesses reported in the literature of both LPS and EVM resulting in the aforementioned novel contribution, while the second level will demonstrate the flexibility of the LPP approach by using it successfully in a very demanding industry given that it previously has been shown to work successfully in shipbuilding. This is discussed more in Section 3.4.

Before continuing it is worth noting that approaches commonly used in manufacturing enterprises, such as value engineering, are often not used in project-based industries such as shipbuilding and pressure vessel fabrication because often the contract is based on a well-developed specification. If a manager wants to improve the project further, he will have to not only prove to the customer that it is better but also do so at the risk of losing progress and spending hours on defining the improved solution.

### 1.2 Method

The case company was chosen because of its genuine attempt to improve performance, and such motivation is paramount for success. In contrast to the shipbuilding case reported by Emblemsvåg [12], this case allowed testing LPP in an even more demanding case. Hence, this study is a natural progression of earlier LPP research.

As such, the overall approach is more inductive than deductive in that a case is used to offer more generalized findings. Through induction, generic theory can be developed over time through a succession of successful cases, as described in Section 3.1, 3.2. To increase the robustness, the literature is used deductively where applicable. Also, the literature offers a starting-point through the EVM- and LPS literature, presented in Section 2.0.

The work presented herein results from implementing LPP through successive projects from early 2018 through late 2019 using the ‘action research’ approach. It is important to note that the case company came out of a large downsizing in 2017 following the offshore crisis in 2015-16, which effectively excluded any thorough planning prior to implementation. Execution time of the subsequent projects was an important parameter for the contracts.

Action research involves the discussion of problems followed by group decisions on how to proceed [13], and this includes the active participation by those who perform the work and contribute to the exploration of problems and alternative solutions. After investigating these problems, the group decides, monitors and analyzes the consequences. Regular reviews of the execution of any decisions follow thereafter. The group decides when a particular plan or strategy has been exhausted or fulfilled and brings any newly perceived problems to discussions. As such, action research may be defined as an emergent inquiry process in which applied behavioral science knowledge is integrated with existing organizational knowledge and applied to address real organizational issues [14].

There are four key aspects of action research that must be handled well to give good results from a epistemological perspective [15]:

1) As action research generates localized theory through localized action, knowledge of context is critical,
2) the quality of relationship between group members and between members and researchers are paramount,
3) the quality of the action research process is grounded in the intertwining dual focus on both the action and the inquiry processes, and
4) The dual outcomes of action research are some level of sustainability (human, social, economic, and ecological), the development of self-help and competencies out of the action and the creation of new knowledge from the inquiry.

All these four aspects are handled well in this case since the action researcher worked in the company as an executive and the employees were fully aligned with the process. However, there are potential caveats as discussed in Section 3.5 since the key action researcher is also an insider.

When it comes to studying the results across the cases, the approach does not lend itself to statistical analyses due to lack of replication because the sales conditions that the projects were sold under differed substantially as did the contracts and contractual parties. In very competitive situations, for example, the projects will be far more difficult to secure leading to very tight budgets. Instead, the changes from case to case were measured and discussed considering the context and knowledge of the employees, as done in Section 3.3.

The analytical approach to support the action research is modeling via the scheduling system implemented in Primavera P6. A model is derivative of a specified analogy, but the model has a more elaborate or "deeper" structure than the analogy [16] where an analogy consists of assertions of similarity or difference between corresponding elements in two different systems, and about the sets of causal relations operating within each system [17]. Clearly, most models are ‘wrong’ or ‘limited’ in some key aspect [18]. Nevertheless, models are the tools of scientific thinking [19]: physical models are tools of descriptive analogical thinking, mathematical models at large are the tools of analogical argumentative thinking. The modeling proposed is therefore a good fit for action research and an argumentative/participative/conversational approach, as found in Section 3.0.

As Stringer and Genat [20] note, action researchers ‘engage in careful diligent enquiry not for the purpose of discovering new facts or revising accepted laws or theories, but to acquire information having practical application to the solution of specific problems related to their work’. In other words, the positivist, reductionist doctrine of epistemology often used in modeling [21] is not applied but rather a participatory, relativist doctrine that used qualitative as well as quantitative tools (see Ref. [22] for more information). The work presented herein can therefore not automatically be transferred to any project-based industry without a clear understanding of any contextual differences.

Finally, generalization to other project-based industries is discussed by inference since the research method is contextual. Given that the production of pressure vessels has many more requirements than any other project-based industries this author is aware of, except production of nuclear pressure vessels, the practical insights and scientific knowledge would be most likely transferable to many other project-based industries when particular contractual issues are properly understood, as well as to the five peculiarities of project-based industries discussed later. Scientific contributions are discussed more in Section 3.4 with limitations being addressed in Section 3.5.

The details of the action research itself are discussed in Section 3.0. Sections 3.3, 3.4 show that EVM and LPS can be successfully combined, also in general (and not merely in this case). Further improvements are identified and discussed in Section 4.0. Concluding remarks are provided in Section 5.0.

## 2 Comparing EVM to LPS – key points

This section provides a brief overview of the key points of the two approaches. Other approaches such as CPM, PERT and Critical Chain are omitted because they are insufficient in themselves to meet the contractual requirements, probably due to their poor link to the financial results of projects [23], and they do not solve the shortcomings of EVM. In fact, EVM, CPM, PERT and Critical Chain are used together by most practitioners although they come from distinctly separate historical developments as explained by Smith [23]. In fact, Smith [24] states explicitly that “The Earned Value Methodology has been a ‘Best Practice’ for monitoring and evaluating project cost performance for over fifty years”. Therefore, only approaches that can *improve* project execution further using EVM as a starting point are included.

Section 2.1 summarizes EVM and can be compared to Section 2.2 and the synergies presented in Section 2.3 that are the basis for LPP.^1^

### 2.1 Brief introduction to relevant EVM key points

**Fig. 1:**
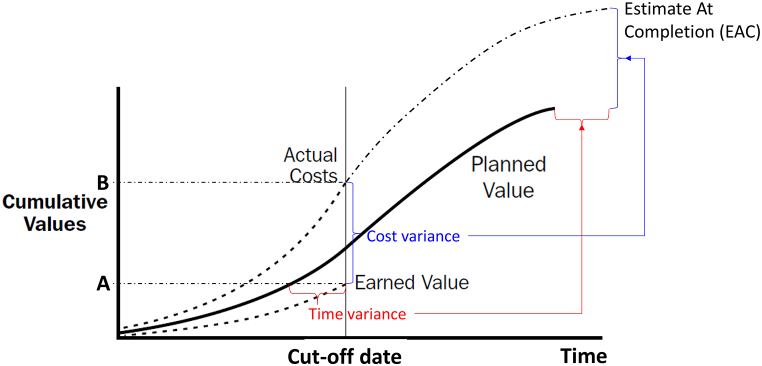
**– Research relevant EVM key principles.** Based on PMI [26].

EVM has a cost-centric approach due to its roots in cost-reimbursable contract environments [3]. This can be best illustrated by the s-curves used, see for example Fig. 5. To explain EVM, conceptually, see Fig. 1. The original plan organized after the Work Breakdown Structure (WBS) will result in the Planned Value (PV). The PMI [26] defines PV as ‘the physical work that is to be performed including the estimated value of this work’, and the WBS is defined as ‘a deliverable-oriented grouping of project components that organizes and defines the total scope of the project’. Hence, the PV is the accumulated values of all the individual WBS in terms of start dates, stop dates, hours used and materials consumed, which is where scheduling becomes key.

A schedule basically defines when and how long work will occur and how each activity is related to the others [27], but the schedule has many applications such as 1) providing a road map for systematic project execution, 2) securing the means by which to gauge progress, 3) identifying and resolving potential problems, 4) promoting accountability at all levels, 5) providing a time sequence for the duration of activities, 6) helping people understand both the dates for major milestones and the activities that drive the schedule, 7) being a vehicle for developing a time-phased budget baseline, 8) enabling a basis for managing tradeoffs between cost, schedule, and scope, 9) Giving decision-support in deciding among possible sequences of activities, 10) determining the flexibility of the schedule according to available resources, 11) predicting the consequences of managerial action or inaction in events, 12) enabling the allocation of contingency plans to mitigate risk, 13) forecasting the effects of delayed, deleted, and added effort, as well as possible avenues for time and cost recovery, and finally 14) verifying and validating proposed adjustments to the planned time to complete. From the history of scheduling [28], we therefore understand that scheduling as a discipline evolves. Note that material is often left outside the scheduling resulting in a focus on hours and sometimes hourly costs if the hourly rates vary significantly in the project. The materials are then handled in a separate procurement plan.

Then, at a certain cut-off date, typically at the end of every week or every month, all project data is reported to the planning system, and the Earned Value as well as various variances are calculated. The PMI [26] defines Earned Value (EV) as ‘the physical work actually accomplished including the estimated value of this work’. In the figure we see that at the cut-off date, the EV is less than the PV. This implies that the work actually accomplished at the cut-off date is less than planned and the dotted line to the value A indicated that the amount of work corresponds to the planned amount of work at some earlier point in time. The difference therefore represents a time variance. This time variance normalized for the PV is then the SPI.

A weakness of EVM is that the lack of process-oriented metrics can lead to poor management due to lagging decisions and ineffectively timed corrective actions [29]. CPM, Critical Chain and PERT offer little help when the number of activities becomes large [23]. This weakness also has spillover effects to costs.

The second dimension of interest is costs. At the cut-off date there is a difference between the actual costs (B), see Fig. 1, spent to achieve the work and the planned costs (A). This is the cost variance, and the CPI is then the cost variance normalized for the actual costs. In this sense, the CPI is a productivity measure and if it is less than 1, the project has a lower productivity than planned and will fail to reach its budget. Similarly, if the SPI is less than 1, the project has slower physical progress than planned and will fail to be delivered on time.

Then, the scheduling system can compute EAC through extrapolation and dependencies between the Work Packages (WP) which consists of a WBS and an activity, as discussed later, provided that no countermeasures for the performance problems are implemented and further performance slippages (i.e., deteriorating CPI and SPI) are stopped. By actively replanning and identifying opportunities for better execution, however, the EAC can become better than shown in Fig. 1. Potential scheduling buffers can be released, which implies that the project reduces its risk buffer(s) on WBS level both in time and costs. Releasing such buffers can help, but normally only if further deterioration of SPI and CPI is stopped.

The experience of this author suggests that delivering on time results in even higher costs and delivering on budget results in even longer delays, but there is a limit. Longer delays can incur extra costs such as financial costs, liquidated damages, interference with other projects, etc. so in real life, the best option is normally to find a compromise in cooperation with the customer. Another experience worth noting is that using dependencies can be difficult, leading to conservative and unrealistic scheduling as noted by Emblemsvåg [12]. Due to the practical problems of using dependencies, such as determining exactly when one activity can commence after another or to what extent they can be executed in parallel, he even discourages it altogether.

Note that the WBS can be reused to some extent between projects even though projects are unique. For example, most bicycles have two wheels but individual bicycles will have different kind of wheels resulting in different costs, different installation details, etc. From a scheduling perspective, the WBS can then be reused but adjusted for different start- and stop dates and potentially different duration- and resource estimates.

### 2.2 EVM versus LPS

The research on LPS started in 1992 during a consulting project and culminated in Ballard's Ph.D. dissertation [30]. It belongs to a larger framework called Lean Construction (LC), which can be traced to the seminal work of Sanvido [31] on systems, Koskela [32] on flow and Laufer and Howell [33] on construction project planning [34]. Conceptually, LC and LPS attempt to bring the benefits of lean manufacturing into project related industries such as the construction industry.

Ballard [30] provides a very good overview of LPS in his PhD dissertation, and he borrows several key ideas from Koskela [32]. First, Koskela [32] questions the very definition of a “project” provided by the PMI [26] and he argues it is misleading because it focuses only on outputs and on an untested assumption of uniqueness. The result is a discouragement of learning from other industries. With this sole focus on output, the physical conversion activities become the focal point while flow- and value activities are ignored, and the end result is a contracting mentality rather than management of production or flow.

Second, project management becomes reactive since control is obtained by detecting negative variances from target, whereas the purpose of the active concept of production planning and -control in manufacturing is to conform to plan [35].

The third key idea from Koskela [36] are the five key principles of operational work management:

1. A job shall not start until all the conditions for execution required for completion of a job are available (a sound job) [30]. The person or group that produces such job assignments is called the “Last Planner” by Ballard and Howell [37].
2. Planned assignments must be actively prepared ahead of execution to create a pull mechanism. The purpose is to avoid large material buffers from emerging on site.
3. If any jobs are not executed, there is an investigation into the causes and corrective measures are put in place to enable continuous improvement.
4. Assignments are measured and monitored using Percent Planned Complete (PPC), which is the ratio (in percentage) between the number of planned activities completed divided by the total number of activities assigned.
5. Avoiding lost production by maintaining a buffer of sound assignments is important.

Item 1 requires additional explanation. The core of the argument is that in project-based industries, or Architectural, Engineering and Construction (AEC) industries as Koskela [32] calls it, the focus on the directly value-added activities (conversion activities) often takes place at the expense of supporting activities that secure information flow and activities that create value for the customer. This is the natural result of the fact that traditional contracts – and contract law – are based on the idea of self-optimization [38]. The result is that the contractual requirements become more important than finding the best possible way, and it leads to a Nash equilibrium where everybody loses [7].

To understand the differences between the two, structure is a good starting point. Conceptually, LPS has three hierarchical planning levels [39]. The *initial planning* provides the project budget and schedule, and it pushes completions and deliveries onto the project. The *look-ahead planning* is pulling resources and further adjusts and details budget and schedules. *Commitment planning* is based on comparing what **can** be done to what **should** be done (detailed in the schedule), and then find the best fit. This is where production controls start based on short-term commitments and follow-up that prevents major deviations from building up. By people committing themselves to the plan, they can also be held accountable.

**Fig. 2:**
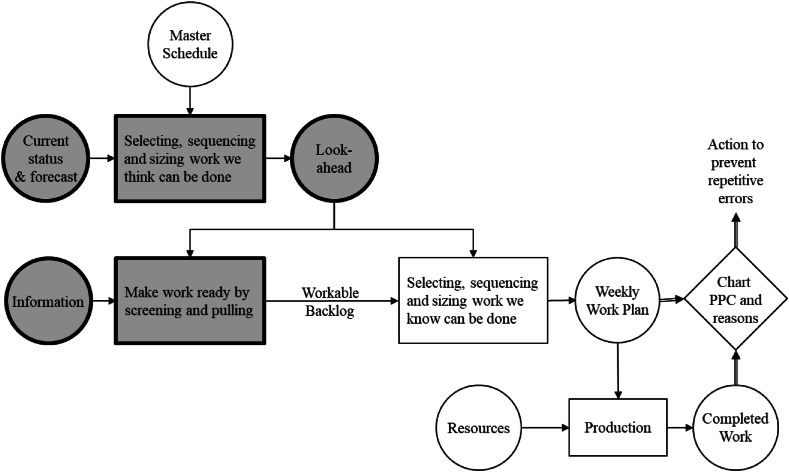
**– Last planner system with look-ahead processes Highlighted.** Source: Ballard [30].

In Fig. 2, the same process is depicted in more details. Crucially, work is to be planned with progressively more details from the top to the bottom in the planning levels. The “Initial planning” is the same as the “Master Schedule”. The “Look-ahead planning” comprises all of the grey boxes in Fig. 2 whereas the rest in Fig. 2 is the “Commitment planning”. There are a number of important aspects of look-ahead planning to be noted [30]:

1. The “Look-ahead plan” is more detailed than the “Master Schedule” by decomposing the activities in the “Master Schedule” into defined Work Packages and jobs. In turn, sequence and rate from the Master Schedule can be improved and adjusted to realities.
2. More detailed methods for executing work are found in the “Look-ahead Planning” and once this is known, workflow and capacities can be matched.
3. The jobs to be assigned, are to be identified 3–12 weeks in advance in the look-ahead process depending on project characteristics such as reliability in the planning system, lead times for acquiring information, materials, labor and equipment. Only jobs that can be completed on schedule are advanced from one week to another and finally into production, which creates a backlog of sound activities.
4. Constraints in the look-ahead process must be analyzed and removed so that the jobs become sound.

There are also many important remarks to make concerning the “Commitment Planning” in which the weekly work plans are made [39]:

1. The assignment must be specific enough to facilitate clear judgment concerning completion or not.
2. The assignments must be sound, i.e., executable – nothing must be missing. This is to ensure flow, and [40] identified seven preconditions that must be satisfied in order for an activity to be sound/executable in production; 1) construction design (information), 2) components and materials, 3) workers, 4) equipment, 5) space, 6) connecting works – previous work and 7) external conditions such as weather, government rules and licenses.
3. The assignments must be sequenced correctly to avoid rework.
4. The assignments and the capacity must be matched.
5. Assignments that are not completed according to plan are analyzed to prevent similar mistakes in the future.

These five items may seem self-evident, but according to Ballard and Howell [39],” … it is by far the exception rather than the rule to find construction contractors that make quality assignments, and even rarer to find engineering firms or departments to do so. Many more or less, pursue a strategy of flexibility, attempting to be prepared for whatever work may become available”. The experience of this author is the same, and failing to plan, coordinate and execute properly is often redressed in the name of ‘flexibility’. It should also be noted that the failure to make quality assignments effectively makes it impossible to pass judgments on both physical progress and remaining work to such an extent that the results are significantly impacted. This opens up for potentially gaming the reported results, which can be tempting if there are significant project deviations. In fact, the construction industry is the industry with the least productivity improvement with just 1 % improvement per year in the global average of the value-added per hour, which is roughly a quarter of manufacturing, according to the consultancy firm, McKinsey [41].

Note that look-ahead planning has essentially a risk- and uncertainty management purpose. However, the philosophy is different. Conventional Project Risk Management (PRM), which can also be integrated with EVM as shown by Babar et al. [42], is based on the ability to analyze the likelihood for something to happen, its impact, then rank it among all other things that can happen and then develop suitable countermeasures. Alternatively, Schedule Risk Analysis can be employed, as illustrated by Song et al. [43]. In look-ahead planning as contemplated in LPS, however, the risk is acknowledged but it is dealt with immediately through the seven preconditions for execution defined by Koskela [40] that determine if it is a risk or not. Essentially, the approach advocated here and as clarified by Emblemsvåg [44], is based on maneuverability and not the ability to analyze as in conventional PRM. In this paper, no attempt is made as to discuss the merit of one over the other, but it is interesting to observe, however, that agility/maneuverability is highly sought after in the modern military doctrine of maneuver warfare [45] and also in agile methods such as Scrum often used in a wide range of settings and for varying purposes, in- and outside of the traditional software development context [46].

**Fig. 3:**
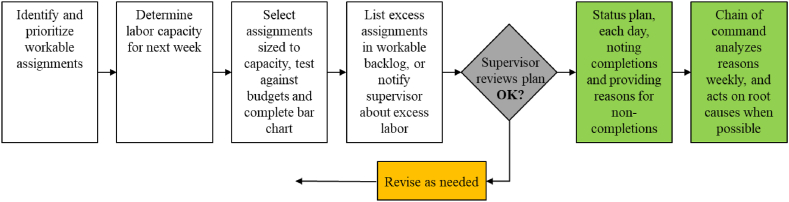
**– Foreman weekly work planning procedure.** Source: Ballard and Howell [39].

For the foreman, the weekly work planning procedure is as illustrated in Fig. 3. The diagram must be read from left to right in a sequential manner. Thus, we see that when the supervisor reviews the plan, there are two outcomes. Either the person is told to revise it, or it follows the flow to the right into execution. Then, the next week the procedure starts over again.

Foremen are key in LPS because the planners are not separated from the doers, which is a common mistake in business in general [47]. The PPC and collection of Reasons for Non-Compliances (RNCs) are the most used metrics in LPS [48]. PPC measures the production planning reliability in short-term plans as the number of commitments successfully accomplished over the total number of commitments made for a specific period [29].

Typically, the PPC on non-lean processes are in the 35 %–65 % range, whereas after LPS is implemented performance typically rises to 75 % - above 90 % [30]. In fact, better than 70 % was very rare prior to LPS [39]. Such results indicate that there are too many performance anomalies in the traditional project management paradigm to accept it, and the subsequent research resulted in the Lean Construction paradigm [34].

From this brief overview, we summarize some crucial differences between EVM and LPS:

1. LPS pull governs the flow of materials and information whereas EVM pushes the release of information and materials [30].
2. LPS obtains control proactively via execution whereas in EVM control is reactively obtained by variance detection and countermeasures [30].
3. Capacity and inventory buffers are used to absorb variation (*mura*), and feedback is included at every level in LPS to ensure system adjustments [30]. This approach does not exist in EVM.
4. LPS tries to mitigate variation in every aspect (product quality, rate of work) and manage the remaining variation, while in EVM variation mitigation and management is not considered [30].
5. Decision making in LPS is distributed while in EVM it is centralized sometimes to one single manager [30].
6. EVM embeds neither the conception of soundness nor backlog [30].
7. LPS production system design resists the tendency toward local sub-optimization [30]; however, EVM promotes optimization of each activity through its reductionist approach. This becomes a challenge for LPS if the contracts are designed for EVM thinking. If LPS is left outside the contracting regime, it can become disconnected and scheduling becomes unduly susceptible to manual judgements as explained by Emblemsvåg [12].
8. EVM focuses on the project macro-level. This is necessary but insufficient. LPS recognizes that any successful project will inevitably involve the interaction between project- and production management [49], which we can generalize to the interaction between project- and functional management.

In summary, from the literature we find that EVM handles issues related to the contract, and it has some very desirable metrics such as the SPI and the CPI, but it fails at a number of important issues including improving project performance, which LPS achieves. Both approaches can be gamed, but in different ways. Hence, combining both to reap the best outcomes is worthy of further consideration.

### 2.3 Merging LPS and EVM into LPP

In project-based industries, the contracting regime is crucial because it determines aspects of how to work [7] and even the WBS in some cases. Therefore, the contract, the WBS and the performance of work are intimately related. This realization from practical experience was one of the motivators of Emblemsvåg [12] for developing Lean Project Planning (LPP) in the first place. Basically, the idea was to identify how the two approaches (LPS and EVM) could reinforce each other. Hence, LPP became essentially a synthesis of EVM and LPS with an explication of planning as a communication process [12].

**Fig. 4:**
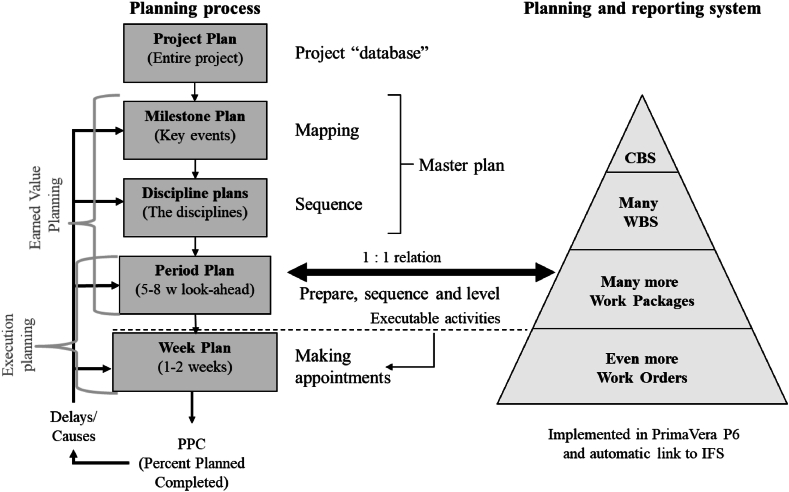
**– Lean project planning overview.** Source: Emblemsvåg [12].

The LPP approach is visualized in Fig. 4. The approach consists of a system part in a software, such as Primavera P6, and a process part that follows the LPS thinking with some EVM elements such as WBS, physical progress, budgets and so on.

**Fig. 5:**
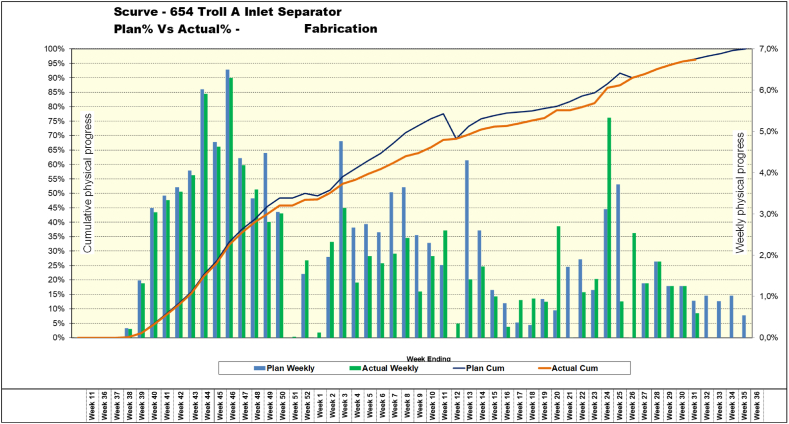
**– The s-curve for weekly- and cumulative physical progress for the Troll A Inlet Separator project.** Note that the project started in Week 11 but for 26 weeks the project waited for ordered materials, which arrived in Week 37. This long lead-time of the procurement process is due to the highly special grade and -quality of the materials used in the pressure vessel.

The Master Plan is from EVM whereas the Period Plan is from LPS, but they are combined through the Period Plan level. The purpose is to look ahead 5–8 weeks, i.e., a given period, and it is updated weekly. The time period is chosen to sufficiently manage or maneuver around most challenges and to avoid administrational burdens that are too significant. Long lead-time items are followed up separately in traditional EVM style. For example, shell strakes and heads for the case company have more than 20 weeks lead time.

Crucially, plans should be detailed while approaching execution and not before [11], which reduces the need for re-planning, and it increases the maneuverability. Furthermore, the 8 weeks duration limit provides very good tracking of physical progress, which enables robust calculation of CPI and SPI. Thus, the EVM becomes more reliable/difficult to influence – an issue voiced by Kim and Ballard [50]. Another crucial aspect of combining EVM and LPS through LPP is to define Work Packages (WP) so that a 1:1 relation is secured between activities in the Period Plan and the WPs. A WP is defined by the WBS, a standard activity and a zone in the large projects. For example, the WBS can be ‘Nozzle 41’ and the standard activity can be to ‘weld’. The WP becomes ‘Weld Nozzle 41’.

LPP adopts the seven preconditions for execution defined by Koskela [40] and used in LPS to ensure quality assignments and controlling WIP. The case company also blocked activities in the time registration system if a precondition for an activity was not fulfilled. This approach instills discipline, prevents early starts and enforces the usage of the seven preconditions.

The lowest level is the Week Plan level, which is essentially a work-list for the foremen and workers which covers the next two weeks. Despite their simplicity, the Week Plans have several important functions:

1. Since the Week Plans are the most operational part of the planning, it is here the communication part mostly takes place where tacit knowledge and a real dialogue create understanding [12].
2. ‘Commitment planning’ also takes place here [39]. In the weekly lean meeting, project participants make appointments as to what to do, when to do it and in what sequence. Possible issues are dealt with right there and then. They commit themselves to each other.
3. The weekly follow-up of these Week Plans in the lean meetings is crucial. Completing the whole sequence of Plan-Do-Check-Act is the crux of planning and control regardless of approach.
4. The PPC metric is also employed in Week Plans to measures performance. A score of 100 % implies that all tasks have been performed as promised.

A final key principle is that those who know the jobs must do the planning [10]. Hence, supervisors are integral to the well-known Plan-Do-Check-Act (PDCA) process. Thus, the planner facilitates the planning process, manages tools, analyses reports and so on – but she does not set dates, define durations and give hour consumption estimates.

The actual combination of EVM and LPS takes place in the planning system on the period plan level where a WP is defined so that it becomes an activity in the Period Plan. Furthermore, several WPs must constitute a complete WBS – there cannot be a many-to-many relationship. On the planning process side, the overall process follows the LPS but with elements from EVM necessary to satisfy the EVM part related to the usage of both WBS and budgets on the WBS level. In terms of reporting, people must for every WBS report physical progress, consumed hours/costs, remaining hours/costs, any changes in dates (i.e., duration) due to deviations, how to close any deviations, status for WPs (addressing the question of whether they have they started/finished is normally sufficient), and PPC for the week plan. Furthermore, they must address many of the same parameters when they look-ahead and plan the next week. A key element is that this entire process takes place in a weekly meeting – typically lasting an hour. The purpose is to facilitate genuine communication where issues are addressed immediately and not through mechanistic reporting of numbers to people who are not involved in the meeting and lacking local information to make intelligent decisions. The exception is issues related to the contract.

## 3 Implementation and results of lean project planning (LPP)

The cases are from Midsund Bruk (MB) in Norway which has delivered a large share of the pressure vessels on the Norwegian Continental Shelf (NCS). The characteristics of this company is a result of its market where key value elements are flexibility, high delivery precision, right quality and no Health, Safety, and Environment (HSE) incidents. The most important characteristics from the perspective of this study are that:

1. The production of pressure vessels is extremely demanding due to its very high, quality requirements in combination with high-grade steel alloys such as 6 Mo and Inconel. Such alloys allow minimal welding mistakes, which in combination with long delivery times and high material costs make such production very unforgiving for mistakes altogether. The quality issues of EVM noted earlier will therefore be potentially devastating for project execution.
2. A limited number of attack-points on the pressure vessels make it difficult to use more people to close any scheduling deviations unlike most other projects such as shipbuilding and civil construction where space is normally ample. The strict welding requirements also imply that securing enough welding capacity is demanding itself due to the many certifications required for each welder.
3. The pressure vessels themselves are relatively small and therefore produced in production lines found in some manufacturing companies, but due to the varying technical requirements, varying dimensions, varying number of interfaces, varying steel grades, etc. the pressure vessels are really small projects that are also organized in teams of people as projects from engineering, procurement all the way to delivery. From a scheduling point of view, this implies that the entire portfolio must be planned simultaneously and not project by project as, for example, Emblemsvåg [12] describes.

In an effort to improve project execution, LPP was implemented in 2018-19. An initial schedule for a project was developed, implemented, the results were studied and a better version was implemented in the next project, and so on. The results were deemed better if the CPI and the SPI approached 1.0 over this two-year period on a weekly basis. Furthermore, all other contractual obligations were to be fully met. This is a clear link to EVM, but at the same time we also measured weekly LPS metrics such as soundness of activities 4 weeks before execution and we conducted lean meetings.

The author of this paper was the General Manager of the company at the time, but he also served as a lean facilitator helping the managers conduct the lean meetings, discussing improvements with the project teams and also resolving disagreements occurring during implementation. The research is therefore very much action research as the researcher and the people doing the actual work were deeply involved. Of course, this makes reliable performance metrics all the more important to avoid biases. The CPI and the SPI metrics help with avoiding biases although they cannot be ruled out completely.

In the next two sections, the implementation of LPP for the first two projects is presented. These two projects were the most demanding from both a technical point of view, and learning point of view.

### 3.1 The Troll A Inlet Separator project

The Troll A Inlet Separator is 14.5 m high, 4.5 m in diameter, built in 126 mm thick P500QT steel and cladded on the inside with 904 Inconel with 7 mm minimum thickness. The mass is about 180 tonnes. The project also included a corresponding 48 inches inlet spool, complete process internals and 24 nozzles. The largest nozzle is 48 inches, then there is 1 of 32 inches, 2 of 10 inches, 1 of 4 inches, 1 of 3 inches and 16 of 2 inches. There are also 2 blinded gangways of 26 inches. Many of these nozzles are measuring levels in the separator, some have jig sets and there are also vortex breakers. The project was planned at 531 days from start to finish (almost 76 weeks), and the delivery marked a milestone in the overall project for Aker Solutions, the EPC contractor, and Equinor and MB.

This project was the first one where LPP was implemented. Since pressure vessels have few attack points and most activities are shorter than the recommended 8 weeks maximum duration, the schedule consisted of many small and clearly defined WPs. Thus, each major weld became a WP, each nozzle became a WP and so on. This resulted in a detailed WBS (118 items) but only 38 WPs covering 509 activities and 838 dependencies. Crucially, the WPs became almost digital concerning their completion (completed or not). Thus, estimating the physical progress became easy and reliable. With suitable dependencies, the schedule also became accurate for costs and duration/time and milestone dates.

From Fig. 5, we see that accurate EAC forecasts were achieved in fabrication. The physical percentage points refer to the amount done in a week, compared to the total project (100 %) or in a cumulative sense. So, if a weekly percentage is 4 %, that means that 4 % of the total fabrication work is to be completed in that week.

Clearly, the execution of the project (orange line/green columns) followed the planned value curve (blue line/blue columns) well. Then, execution ran into a period of some major technical challenges because of failing cladding equipment that literally burnt up when cladding the deep and narrow 2-inch nozzle holes. Finally, equipment was obtained for handling the high temperatures. As a consequence of the time this took, in combination with many Variation Orders (VORs) in the project discussed in the next section, as well as a variation order of this project, the entire project portfolio had to be readjusted. Hence, the project was re-baselined to use the summer of 2019 to maintain the planned delivery date. It was delivered 2 days ahead of schedule.

The CPI, however, took a hit due to the spiral cladding equipment failure. Despite maneuvering well, by putting people on relatively productive tasks, such ‘firefighting’ cannot solve the problem of failing equipment. Once the new equipment was up and running, the CPI improved.

It took training on the part of the organization to make look-ahead planning work well, and everything was planned on days to provide enough clarity for accuracy and learning. With reference to Table 1, performance was quite poor in the beginning with too many WPs being unresolved by the time of execution, which can be seen since not all the seven conditions for execution are satisfied indicated by a zero (0). The causes are listed to the right in Table 1.

**Table 1:**
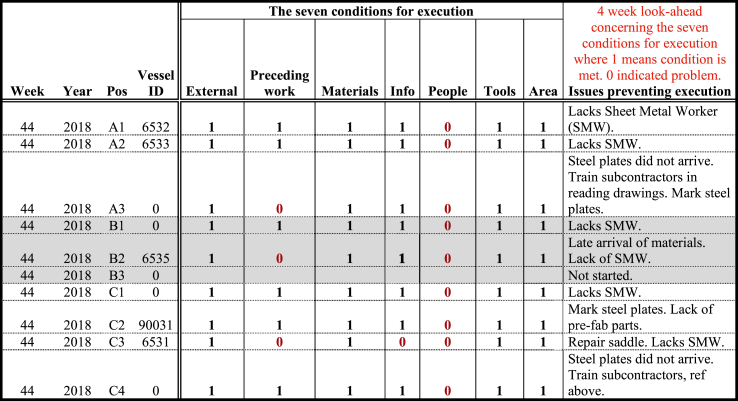
– The 4-week look-ahead plan for Week 44 for the project portfolio, 2018.

Yet, from Fig. 5, it is clear that this did not give many problems early on due to the semi-automatic nature of sub-arc welding of the shell strakes of the pressure vessel body. However, later in the project, when coordination became a greater issue due to a large number of details that must be executed in an exact order/sequence to avoid quality mistakes, the ability to follow the schedule varied. In subsequent projects, the performance improved significantly. Hence, it takes time for real organizational learning to take place.

There were several other challenges to address. First, conducting the lean meetings effectively and efficiently was a challenge on top of the operational issues just discussed. Principally, the organization needs to learn the importance of making- and keeping quality commitments [39]. Management of these meetings is key.

The solution to this was to train people to communicate clearly, ask questions and provide constructive feedback so that they could make quality commitments. In many lean contexts, visual means are often employed, but in a complex project schedule that depends on the overall project portfolio schedule, visual means simply do not work.

Second, the person in charge of the lean meetings had to help people improve. For example, far too often discussions turn away from execution towards engineering details. This is important to stop unless it is a quick, engineering clarification.

Third, approaches for looking forward had to be taught so employees would stop focusing on explaining why things did not work. This is partly a matter of communication but also attitude and habit. In general, it takes effort to learn the language of planning and communicate about it. However, as people realized that the LPP system actually resulted in better plans and better performance the interest in planning greatly improved. As such, the improvement process became self-reinforcing, which is important for the process to sustain itself over time. The next project offered more challenges, but is presented anonymously at the request of the end-client.

### 3.2 The large separator package project

**Fig. 6:**
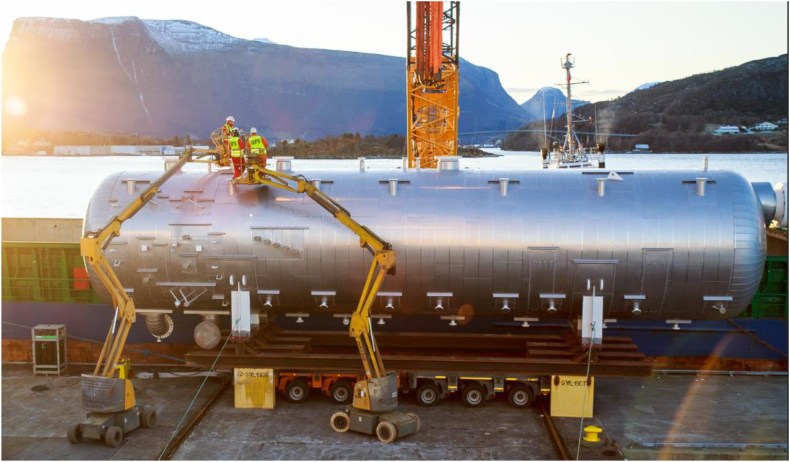
**– Shipping the last pressure vessel on quay.** Photo by Jiri Paur.

The project had 8 pressure vessels, and LPP was implemented using a schedule for every vessel individually and a schedule for the project in total, which allowed scheduling and follow-up of each pressure vessel individually and the project in total. The project was delivered on time. The last pressure vessel was delivered 3 days ahead of schedule, and shipped thereafter as shown in Fig. 6. This was all the more impressive because the project had a large number of VORs, increasing the contract by approximately 25 %, despite that the 4 largest pressure vessels were supposed to be copies of an earlier project. Additionally, a known, new factor of third-party contractors had to be included.

From an action research perspective, the results from the Troll A project were incorporated. As before, the WBS elements were standardized across projects, but the number of WPs and dependencies varied. In this specific project there were 365 WPs, 2503 activities and 4740 dependencies. Thus, the schedule was large for a pressure vessel project.

**Fig. 7:**
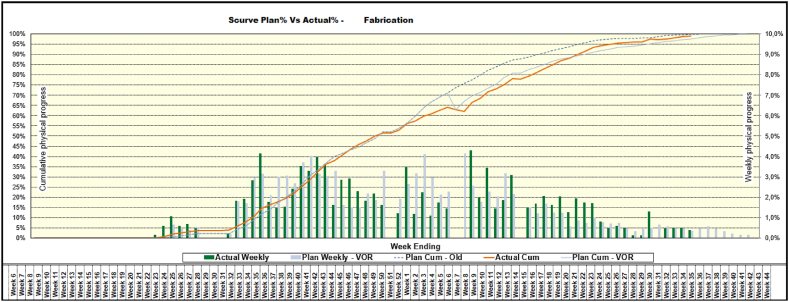
– The s-curve for weekly- and cumulative physical progress for the separator package project. Same axis interpretation as before.

Fig. 7 shows that despite the increased complexity, the performance was very good. The EAC and delivery time was estimated after 50 % progress with great accuracy (about 1 % deviation) despite VORs entering the project at various stages during the project execution. Note that the EACs could have been estimated accurately earlier had it not been for all the VORs that came the first 6 months of execution. This is in line with the findings from Fleming & Koppelman (2005) that after 20 % progress EACs can be reliably forecasted.

Due to its sheer complexity, heavy involvement of third-party subcontractors and a large number of VORs, the LPP implementation was stress-tested. Thus, this project was highly valuable from an improvement perspective. Despite the good performance, we identified another four issues to improve.

First, the communicative aspect of planning cannot be overemphasized, and it became even more challenging due to the third-party subcontractors. The solution was to include them as if they were employees. The armlength distance principle often used in procurement and contracting is therefore not useful for project execution. To be more precise, the armlength distance is useful commercially, but a problem operationally. With the importance of contracts in project-based industries and all the problems that follows, as pointed out by Emblemsvag [7], we understand that a contract can be a double-edged sword that must be managed wisely.

Second, the WBS and the clocking structure (activity names and IDs of activities) must be aligned in future projects (the WBS structure) to reduce administrative burden. Unfortunately, this could not be solved for this project, but it was solved for the next project.

Third, managing the subcontractor capacities well to avoid idle-time and extra costs turned out to be demanding. Thus, subcontractors reduce the risk of capacity imbalance, but they also introduce operational risks. One way to manage this situation is to rapidly determine their skill level by developing some standard tests that they had to perform within the first two days on premises. Multi-skilled people could therefore be identified. Another approach was to be conservative concerning the number of subcontractors, something that was realized towards the end of the project when the workload was falling. Finally, the action research sequence can be accelerated by determining whether the improvements are systemic or managerial. Managerial improvements are those that related only to how work is being done, and such improvements can be implemented immediately and not by waiting for the next formal debriefing. This gave many more improvement cycles.

Systemic improvements, however, are those that relate to more fundamental issues such as IT infrastructure, investments in machinery and so on. To incorporate such improvements, typically requires that we start on a new project with fresh schedule and sometimes also investments. Hence, many of the results from these two projects were implemented during the projects but one systemic improvement, solving the WBS and the clocking structure misalignment, remained for future projects.

### 3.3 Business results

Identifying the results of new initiatives is always uncertain because nobody knows what an alternative future would hold. However, given these complex projects and the fact that both were delivered on time despite a number of challenges indicates that performance was undeniably good. In this instance, improved project planning contributed to this good performance. More specifically, the EACs were excellent, and later projects had similar performance. Furthermore, people also found LPP very useful for capacity planning, handling VORs, sequencing activities and maintaining contractual milestones.

Crucially, effective communication in the planning process is key. Communication was improved compared to the situation before LPP was implemented, but communication is also an area of future improvement. The positive aspect of LPP in this regard is that LPP provides a common basis for understanding current performance both through the LPS metrics such as PPC and soundness of jobs, but also by using the look-ahead planning system and the weekly commitment planning meeting (lean meeting) to identify ways to improve. The s-curves, SPI and CPI from EVM are also effective, because we were able to trace these key metrics down to every WBS, teams and weeks. Clearly, these systems reinforce each other, and the result is better control and more effective improvement of performance through effective communication and execution.

However, there is still room for improvements as discussed later. This can be illustrated by the fact that the PPC was around 50 % due to the issues discussed earlier. It should be noted that the project delivered at the time of concluding this research was delivered on time, in less than the budgeted number of hours and without a single quality issue.

### 3.4 Contributions

The LPP system accurately estimated EACs months prior to physical completion. Concerning hours consumed, the actual hours consumed deviated by less than a few percentages from the estimates provided early in all cases. Such level of accuracy was achieved by combining LPS and EVM. Thus, the main scientific contribution is that while LPS and EVM are often portrayed as competitors in the literature, this work indicates that these two approaches are better viewed as complimentary. EVM lacks the process perspective that LPS offers, and LPS lacks the contract-oriented perspective that makes EVM often preferred in contracts. Simply stated; LPS makes EVM work, and EVM makes LPS contractually acceptable. Thus, we find a positive answer to the first research question.

An interesting and recent study in this respect is provided by Lagos and Alarcón [51], where they study how the assessment of the PPC average, PPC deviation and RNCs across project execution can help to evaluate the project expected schedule accomplishment. After studying 25 projects, they concluded that the projects that maintained a PPC equal to or higher than 75 %, with a PPC standard deviation lower than 11 %, were likely to obtain a lower schedule deviation and, thus, had a higher probability of success, which increased significantly when reaching accumulated PPC averages higher than 85 %.

As noted earlier, the causalities are difficult to judge without project insights – did the project do well because it was relatively better/more realistically planned upfront, hence making commitments easier to maintain, or did the PPC actually help? In this study, through action research, we had the benefits of being insiders from sales through execution, and it is no doubt that the LPP improved performance significantly. That being said; it is crucial to perform a proper project review before signing any contract or making any firm commitments impacting schedule and cost. Such risk factors can have major impact on the outcome as discussed by Ibrahim et al. [52]. This topic, however, is outside the scope of this paper.

Both of the projects discussed in this paper were sold under difficult market conditions hence making budgets and plans very tight in terms of both costs and time. Yet, not only did we reach the EACs but we also maneuvered and assumed large amounts of VORs, and still managed to reach the revised EACs. Thus, this study supports the findings of Lagos and Alarcón [51] that the systematic approach to planning and control with the use of process-oriented methods to prepare, execute and control work managed by commitments helped in making EVM work and the projects succeed. This finding also supports Ballard and Tommelein [53] who also stress the importance of process-oriented methods to prepare, execute and control work managed by commitments.

Naturally, more cases are required to determine whether this finding is generalizable beyond the projects discussed here, and to some extent also the projects presented by Emblemsvåg [12]. Although LPP worked well in the cases documented so far, this does not imply that these improvements could not have been achieved in any other way.

The second contribution is to demonstrate that using dependencies can work well if they connect sound activities. In fact, when it comes to LPP itself, dependencies have been discouraged as noted earlier with the result that SPI could not be calculated and forecasts such as EACs were made manually. This discouragement seems premature based on the findings here. It should be noted that this contribution did not follow a specific research question, but it came more from the insights developed during the study.

A third contribution towards the LC literature is to demonstrate that LPS can be merged with EVM without losing its strengths. Essentially, this is the flipside of the first contribution, and it relates to the first research question. Indeed, the LPP version of LPS can make LPS more attractive for lawyers and executives who are predominantly focusing on the contract and its execution. Thus, we can conclude that we offer a positive answer to the first research question.

Furthermore, expanding LPP outside shipbuilding is a contribution addressing the second research question, but perhaps equally important; LC thinking has not been used in production environment similar to pressure vessels to the knowledge of this author, which represents a contribution to the wider LC literature.

### 3.5 Limitations

Care should always be exercised in research with limited sample sizes. In total, 14 projects have used LPP at the case company, and the project planning performance has improved steadily. Furthermore, these projects are relatively small compared to shipbuilding projects, but due to the very few attack points on pressure vessels, deviations will easily have far greater impact on the project execution in pressure vessel projects than in shipbuilding projects. We therefore conclude that also the second research question has been positively answered despite the limited number of cases.

Whether or not LPP can be applied to the wider project-based industries such as civil construction, is still an open question, but we can address such generalizations through inference. Unlike shipbuilding and fabrication of pressure vessels which use the same site in a more manufacturing type of production, civil construction is truly site independent. However, the consequences of having to construct something where it will be used is far smaller conceptually than it may appear.

The main consequences of site construction are fourfold. First, weather becomes an issue, which is partially also the case in shipbuilding but rarely a problem in fabrication of pressure vessels. Second, advanced machine centers are not used unlike in shipbuilding and fabrication of pressure vessels. Third, both shipbuilding and fabrication of pressure vessels require accurate portfolio planning due to the usage of the same machine centers and sites. Fourth, the competition is global whereas in civil construction the competition is at best regional (such as northern-Europe). However, none of these differences imply that what works in one should not work in another. In fact, the fabrication of pressure vessels is the type of production with the most constraints and the highest demands as discussed earlier. Thus, by inference, if project scheduling works in fabrication of pressure vessels, it should also work in civil construction. Obviously, this inference should be tested through future empirical studies before drawing concrete conclusions.

In fact, it is not the type of industry that is perhaps the real challenge, but the type of work. For example, knowledge work, in general, may indeed require completely different approaches. As Ballard and Howell [39] note, quality assignments are rare in engineering, which is also the experience of this author. This paper has focused on the overall project and mostly focused on the production part, but how to improve engineering scheduling, for example, seems to be a natural extension of the research presented herein. Engineering scheduling would also have a major impact on procurement scheduling, which is much easier in pressure vessel fabrication than in shipbuilding due to the sheer amount of procured items.

The final limitation to discuss is the execution of the research itself. As noted in Section 1.2, there is good reason to believe that being a company insider solves many of the typical action research challenges an outsider faces. However, there are some challenges to discuss related to biases in the inquiry process as well as in the interpretation of the outcomes. The interpretation of outcomes is well supported by the performance metrics used, and since these metrics were calculated by a separate person in the company organized directly under the researcher, and not under production management, the biases here should be minimal. Also, the site representatives of the customers help in this respect to identify what the realities are, and there is very little disagreement.

The inquiry part of the action research process, however, is far more difficult to assert because as an insider there are a number of issues to be aware of and the only outsiders relative to the plant did not have access to all the details. The concept of ‘social situatedness’ originally put forward by Vygotsky [54], and situatedness in terms of learning developed by Lave and Wenger [55] is a good starting point, and the key is that the development of individual intelligence requires both social and cultural influences, and the multiple perspectives needed for understanding are provided by context [56].

Being an insider is known to have many advantages [56]: An insider is in a unique position to study a particular issue in depth and with special inside knowledge about that issue, easy access to people and information that can further enhance that knowledge, being in a position to investigate and make changes to a practice situation, challenge the *status quo* from an informed perspective, and an advantage when dealing with the complexity of work situations. In fact, Raelin [57] finds a growing body of evidence suggesting that work-based projects may prove immensely beneficial to the long-term success of companies.

Being an insider also comes with a host of challenges as described by Fleming [58]. Indeed, there are several research traditions that are critical to insider research [56], and they do have valid points to make concerning questions about insider bias and validity [59]. However, there are many steps an insider can take to guard against bias in the work, for example careful attention to feedback from participants, initial evaluation of data, triangulation in the methods of gathering data and an awareness of the issues [56]. A useful solution is to use a ‘critical friend’ who can interrogate and challenge the assumptions of the insider [58].

To minimize the insider issues in this research, the scheduling system was used with separate reporting lines for the project planner than for production management and engineering management. Furthermore, honest feedback from the foremen and site representatives from the customers were elicited concerning the functionality and performance to facilitate triangulation. A planning expert from the mother company was also heavily involved and served as a ‘critical friend’. Finally, two master students from outside were also free to roam around, ask questions and do their own inquiries concerning issues that were adjacent to the project. They also asked questions about the presented research. All these approaches should reduce the insider risks. Overall, however, we believe that the advantages of insider knowledge outweigh the negative risks.

## 4 Future work

A number of future improvements are identified. First, the importance of training cannot be overestimated, as noted earlier. This concerns scheduling in general, but also how to conduct look-ahead planning effectively, make commitments that can be held and ultimately communicate effectively. Regardless of what planning approach a company uses, there is a significant human component that must not be neglected. One part is a matter of training, as noted, but another part is a matter of attitude. The latter is beyond the scope of this work to address, but it is probably critical and can be influenced through recruitment and leadership.

Second, the execution of lean meetings must be improved, because those meetings are the arenas where the aforementioned matters come together and produce a whole. The work of Kjersem [60] provides a good start. She has identified how these meetings can be best structured and managed in shipbuilding projects.

Third, the scheduling system must be linked to procurement, engineering design and other processes. Indeed, creating such linkages are conceptually similar to what has been shown in this paper. Whereas engineering is arguably more difficult to schedule than procurement, both types of work are inherently difficult to assess regarding a number of EVM parameters such as physical progress, remaining work and duration. Therefore, operationalizing such a complete scheduling system in real life is more difficult than production-related work.

Finally, the usage of dependencies in the scheduling must be evaluated and improved. Like the aforementioned issue, using dependencies is conceptually simple, but difficult in real life due to the number of judgments to make. However, it is undoubtedly true that with a good project planning system, as presented here, it is easier to realistically implement dependencies.

Further digitalization of the entire project-delivery system is also desirable. For example, it may be possible to use big data to calculate the heuristics necessary for improving the manual judgements made today when using dependencies, and Internet of Things (IoT) may be possible to use to capture process performance data of various kinds.

It should also be mentioned that Khesal et al. [61] have presented an interesting combination of EVM and Taguchi Methods to ensure the best possible quality execution. This approach should be studied in the future as it would provide significant improvement if it can improve the robustness of project scheduling in an elegant and simple way that also complies to contractual realities.

The graphical approach discussed by Acebes et al. [62] is also interesting because graphical presentation of information is a well-known lean approach that should fit well into the overall LPP concept. What it offers in addition to the software already used, Primavera P6, remains to be seen.

## 5 Concluding remarks

The paper investigates how EVM and LPS can be combined in a synthesis labeled Lean Project Planning (LPP). The purpose is to secure contractual obligations for both the customers and the case company as demonstrated in two real-life cases. This is not to claim that LPP is *the* best approach, but it has certainly resulted in improved results for the companies involved.

However, LPP is no silver bullet. There is still need to train people in scheduling, look-ahead planning, and making quality commitments through effective and efficient communication and managing lean meetings, in particular. Yet, despite these issues, the paper shows that the planning and execution using LPP through successive iterations of PDCA improves performance. This is a step forward because planning has become a living process and not merely steps leading up to a static plan that is outdated quickly. In the wise words of Dwight D. Eisenhower:

Plans are nothing; planning is everything.

## Data availability

The data used in this paper is confidential and cannot be shared.

## Ethics statement

Review and/or approval by an ethics committee was not needed for this study because it did not involve any vulnerable participants, and data was anonymized upon request. Informed consent was provided by participating individuals.

## CRediT authorship contribution statement

**Jan Emblemsvåg:** Writing – review & editing, Writing – original draft, Visualization, Validation, Supervision, Software, Resources, Project administration, Methodology, Investigation, Formal analysis, Data curation, Conceptualization.

## Declaration of competing interest

The authors declare that they have no known competing financial interests or personal relationships that could have appeared to influence the work reported in this paper.

## Footnotes

1 Note that readers not versant in EVM will benefit from the basic introduction offered by Lukas [25], or an advanced presentation by Fleming and Koppelman [4].

## References

[1] Nieto-Rodriguez A. (2021). The project economy has arrived. Harv. Bus. Rev..

[2] Powner D.A. (2008).

[3] Sumara J., Goodpasture J. (1997). Project Management Institute 28th Annual Seminars & Symposium.

[4] Fleming Q.W., Koppelman J.M. (2005).

[5] Yong-Woo K., Ballard H.G. (2000). Proceedings of the 8th Annual Conference of the International Group for Lean Construction.

[6] US DOE (2018). United States Department of Energy, Office of Project Management Oversight and Assessments.

[7] Emblemsvag J. (2020). On Quality 4.0 in project-based industries. The TQM Journal.

[8] de Souza L.F.C., de Souza A.D., Latifi S. (2019). 16th International Conference on Information Technology-New Generations (ITNG 2019).

[9] Koskela L., Howell G., Ballard H.G., Tommelein I.D., Best R., de Valence G. (2002). Design and Construction: Building in Value.

[10] Ballard H.G., Howell G.A. (2003). Lean project management. Build. Res. Inf..

[11] Ballard H.G., Tommelein I.D., Koskela L., Howell G.A., Best R., de Valence G. (2002). Design and Construction: Building in Value.

[12] Emblemsvåg J. (2014). Lean project planning in Shipbuilding. Journal of Ship Production and Design.

[13] Adelman C. (1993). Kurt lewin and the origins of action research. Educ. Action Res..

[14] Shani A.B.R., Coghlan D. (2021). Action research in business and management: a reflective review. Action Res..

[15] Shani A.B.R., Pasmore W.A., Coghlan D., Shani A.B.R. (2016).

[16] Mellor D.H. (1968). Models and analogies in science: duhem versus campbell?. Isis.

[17] Hesse M.B. (1963).

[18] Grosslight L., Unger C., Jay E., Smith C.L. (1991). Understanding models and their use in science: conceptions of middle and high school students and experts. J. Res. Sci. Teach..

[19] Del Re G. (2000). Models and analogies in science. International Journal for Philosophy of Chemistry.

[20] Stringer E., Genat W.J. (2004).

[21] Barlas Y. (1996). Formal aspects of model validity and validation in system dynamics. Syst. Dynam. Rev..

[22] Koshy E., Koshy V., Waterman H., Koshy E., Koshy V., Waterman H. (2011). Action Research in Healthcare.

[23] Smith K.F. (2022). Traveling the critical path: observations & A-musings of an itinerant project management. PM World Journal.

[24] Smith K.F. (2022). Monitoring & analyzing project costs: PMBOK+PLUS tools & templates to facilitate financial analysis. PM World Journal.

[25] Lukas J.A. (2012). Project Management Institute (PMI) Global Congress 2012—North America.

[26] (2008). PMI, *A Guide To the Project Management Body of Knowledge (PMBOK Guide)*.

[27] Gao U.S. (2015).

[28] Weaver P. (April 2006). myPrimavera Conference.

[29] Sarhan S., Fox A. (2012). Performance measurement in the UK construction industry and its role in supporting the application of lean construction concepts. Australasian Journal of Construction Economics and Building.

[30] Ballard, H.G., The last planner system of production control, in *Faculty Of Engineering*. 2000, The University of Birmingham: Birminghan.

[31] Sanvido V.E. (1984). Department of Cicil Engineering.

[32] Koskela, L., *Application Of the New Production Philosophy to Construction*. 1992, Stanford University, Center for Integrated Facility Engineering: Stanford, CA.

[33] Laufer A., Howell G.A. (1993). Construction planning: revising the paradigm. Proj. Manag. J..

[34] Ballard H.G., Howell G.A. (2004). Competing construction management paradigms. Lean Constr. J..

[35] Koskela L., Huovila P., Anumba C. (1997). Concurrent Engineering in Construction CEC97.

[36] Koskela L., Tommelein I.D. (1999). Proceedings of the 7th Annual Conference of the International Group for Lean Construction.

[37] Ballard H.G., Howell G.A. (1994). Proceedings of the 2nd Annual Conference of the International Group for Lean Construction.

[38] Tvarnø C.D. (2013). Behavioral Analysis Applied to Economics and to Law.

[39] Ballard H.G., Howell G.A. (1998). Shielding production: essential step in production control. Journal of Construction Engineering and Project Management.

[40] Koskela L. (2000). VTT Publications 408.

[41] The Economist (2017). Efficiency eludes the construction industry. Economist.

[42] Babar S., Thaheem M.J., Ayub B. (2017). Estimated cost at completion: integrating risk into earned value management. J. Construct. Eng. Manag..

[43] Song J., Martens A., Vanhoucke M. (2022). Using earned value management and schedule risk analysis with resource constraints for project control. Eur. J. Oper. Res..

[44] Emblemsvåg J. (2017). Handling risk and uncertainty in project planning. The Journal of Modern Project Management.

[45] Lind W.S., Colonel Nightengale K., Captain Schmitt J.F., Colonel Sutton J.W., Lieutenant Wilson G.I. (1989). The changing face of war: into the fourth generation. Mar. Corps Gaz..

[46] Hron M., Obwegeser N. (2022). Why and how is Scrum being adapted in practice: a systematic review. J. Syst. Software.

[47] Sussland W.A. (2002). Connecting the planners and doers. Qual. Prog..

[48] Daniel E.I., Pasquire C., Dickens G. (2015). The 23rd Annual Conference of the International Group for Lean Construction.

[49] Abdelhamid T.S., El-Gafy M.A., Salem O. (2008). Lean construction: fundamentals and principles. The American Professional Constructor Journal.

[50] Kim Y.-W., Ballard H.G. (2000). Proceedings of the 8th International Group for Lean Construction Conference.

[51] Lagos C., Alarcón L.F., Tommelein I.D., Daniel E. (2020). 28th Annual Conference of the International Group for Lean Construction (IGLC28).

[52] Ibrahim M.N., Thorpe D., Mahmood M.N. (2019). Risk factors affecting the ability for earned value management to accurately assess the performance of infrastructure projects in Australia. Construct. Innovat..

[53] Ballard H.G., Tommelein I.D. (2016). Current process benchmark for the last planner system. Lean Constr. J..

[54] Vygotsky L.S., Kozulin A. (1988). Thought and Language.

[55] Lave, J. and E. Wenger, *Situated Learning: Legitimate Peripheral Participation*. 1991, Cambridge: Cambridge University Press.

[56] Costley C., Elliott G., Gibbs P., Costley C., Elliott G., Gibbs P. (2010). Doing Work Based Research: Approaches to Enquiry for Insider-Researchers.

[57] Raelin J.A. (2008).

[58] Fleming J. (2018). Recognizing and resolving the challenges of being an insider researcher in work-integrated learning. International Journal of Work-Integrated Learning.

[59] Lawrence B., Murray L., Lawrence B., Murray L. (2000). Practitioner-based Enquiry: Principles for Postgraduate Research.

[60] Kjersem K. (2020). Department of Logistics.

[61] Khesal T., Saghaei A., Khalilzadeh M., Galankashi M.R., Soltani R. (2019). Integrated cost, quality, risk and schedule control through earned value management (EVM). J. Eng. Des. Technol..

[62] Acebes F., Pajares J., Galán J.M., dolfo López-Paredes (2013). Procedia - Social and Behavioral Sciences.

